# Improvements of Motor Performances in the *Drosophila LRRK2* Loss-of-Function Model of Parkinson’s Disease: Effects of Dialyzed Leucocyte Extracts from Human Serum

**DOI:** 10.3390/brainsci10010045

**Published:** 2020-01-14

**Authors:** Andrea Diana, Maria Collu, Maria Antonietta Casu, Ignazia Mocci, Miguel Aguilar-Santelises, Maria Dolores Setzu

**Affiliations:** 1 Department of Biomedical Sciences, University of Cagliari, 09042 Monserrato, Italy; mcollu@unica.it; 2 CNR Institute of Translational Pharmacology, 09010 Pula/Cagliari, Italy; mariaantonietta.casu@ift.cnr.it (M.A.C.); ignazia.mocci@ift.cnr.it (I.M.); 3 National School of Biological Sciences, IPN, Mexico City 07738, Mexico; maguilarsa@ipn.mx

**Keywords:** *Drosophila melanogaster*, *LRRK2* loss-of-function, Parkinson’s disease, motor functions, human dialyzed leukocyte extract

## Abstract

Within neurodegenerative syndromes, Parkinson’s disease (PD) is typically associated with its locomotor defects, sleep disturbances and related dopaminergic (DA) neuron loss. The fruit fly, *Drosophila melanogaster* (*D. melanogaster*), with leucine-rich repeat kinase 2 mutants (*LRRK2*) loss-of-function in the WD40 domain, provides mechanistic insights into corresponding human behaviour, possibly disclosing some physiopathologic features of PD in both genetic and sporadic forms. Moreover, several data support the boosting impact of innate and adaptive immunity pathways for driving the progression of PD. In this context, human dialyzable leukocyte extracts (DLE) have been extensively used to transfer antigen-specific information that influences the activity of various immune components, including inflammatory cytokines. Hence, the main goal of our study was to ascertain the therapeutic potential of DLE from male and female donors on *D. melanogaster LRRK2* loss-of-function, as compared to *D. melanogaster* wild-type (WT), in terms of rescuing physiological parameters, such as motor and climbing activities, which are severely compromised in the mutant flies. Finally, in search of the anatomical structures responsible for restored functions in parkinsonian-like mutant flies, we found a topographical correlation between improvement of locomotor performances and an increased number of dopaminergic neurons in selective areas of *LRRK2* mutant brains.

## 1. Introduction

Parkinson’s disease (PD) is a chronic, multifactorial and genetically heterogeneous neurodegenerative disorder with a tremendous epidemiology that accounts for 1–2% of incidences in the population over the age of 60 years [1], with a peak of 3.5% at ages 85–89 [2,3]. It is distinguished by integrated motor symptoms, namely, rigidity, bradykinesia, resting tremors and postural instability. Although it is perceived as a movement disorder, it is often accompanied by a plethora of nonmotor symptoms, including the decline of cognition, memory, visuospatial functions, anosmia, sleep disturbances and depression, ultimately leading to dementia. Its etiopathogenesis is still controversial, since most cases seem to be sporadic or related to environmental toxin exposure [4,5,6,7,8]. Only up to 10% of PD cases are clearly associated with monogenic mutations that involve 15 contributing genes responsible for familial forms and cause early, juvenile and late onset [9]. Among these [10], the leucine-rich repeat kinase 2 (*LRRK2*) gene contains multiindependent domains that are widely expressed within different brain areas, such as the cortex, striatum, hippocampus and cerebellum, and in dopaminergic neurons of the substantia nigra [11,12,13,14]. Notably, *LRRK2*-PD bears the pathological phenotype that mostly overlaps with the sporadic form of the disease. The discovery of mutations in the *LRRK2*-encoding gene has disclosed novel perspectives in PD genetics [15,16,17,18]. In fact, the *LRRK2* G2019S mutation is more diffuse in Caucasian individuals, including up to 2% of sporadic cases of PD [19,20]. *LRRK2* is a large protein implicated in both guanosine triphosphate (GTP)-ase and kinase activities that controls several signal transduction cascades necessary for mitochondrial metabolism; synaptic vesicle trafficking associated with endocytosis; retromer complex modulation; and, finally, autophagy [21,22].

With regard to the PD genetic approach, the *Drosophila melanogaster (D. melanogaster)* fly represents an extremely powerful model for studying neuronal dysregulation, providing a paramount tool for the dissection of molecular events in the neurodegenerative disease’s aetiology and progression [23].

In addition, and with respect to the *LRRK2* gene, *D. melanogaster* mutants that overlap significant features with the human condition have boosted several studies in PD research because of their alternative strategies for disease treatment [24,25,26] by means of both mutants’ *gain-of-function* and *loss-of-function* kinase 2 domains [27,28,29]. In particular, the missense G2385R substitution within the WD40 domain severely affects autophosphorylation activity [30], leads to a partial loss-of-function of *LRRK2*, and is pathologically relevant for PD, being associated with an increased risk of developing idiopathic PD among Chinese and Korean ethnicities [31,32]. Interestingly, the kinase activity down-regulated in such mutants can be restored by over-expressing the gain-of-function mutation of the gene [33].

Moreover, previous reports [34,35] have sustained the validity of the *LRRK2* WD40 *loss-of-function* (*LRRK2*^WD40^) as an animal model of parkinsonism in *D. melanogaster*.

Several reports have pointed out how innate and adaptive immunity pathways can sustain the progression of PD. However, the real impact of activated glial cells and inflammatory molecules in terms of neurodegeneration or neuroprotection is still under debate [36]. Moreover, the infiltration of circulating T lymphocytes into the brain can produce dramatic effects, since in vivo blood–brain barrier (BBB) dysfunction has been documented as a triggering event in parkinsonian patients [37]. This experimental evidence that highlights the importance of an intact BBB has been confirmed in the last decade by several papers, as recently reviewed by Sweeney MD et al. [38].

Therefore, in order to elucidate the potential impact of anti-inflammatory molecular signals released by T cells, we have tested dialyzable leukocyte extracts (DLE) as a means of alternative direct delivery to transfer antigen-specific information for cell-mediated immunity (CMI). This is the rationale underlying the phrase “transfer factor” (TF) to describe this pool of molecules. Since the pioneering work of Lawrence [39], TF has been prepared by disrupting lymphocytic plasmalemma and subjecting the recovered lysates to dialysis for collecting the biological fractions with molecular weights (MWs) less than 12 kDa. For many years, DLE have been used successfully as an adjuvant or primary therapy, not only for viral, parasitic, fungal and bacterial infections but also for immunodeficiencies, neoplasia, allergies and autoimmune diseases, despite the fact there is no clear understanding of the molecular mechanisms driving the beneficial effects in experimental and clinical studies [40]. Moreover, there is no report yet dealing with possible modulatory effects in the central nervous system being affected by neurodegenerative syndromes with an important inflammatory component, such as PD. Thus, the aim of this paper was to investigate the therapeutic potential of DLE on *D. melanogaster LRRK2 loss-of-function* variants, compared to *D. melanogaster* wild-types (WTs, Canton-S), in terms of physiological parameters that are severely compromised in the mutant flies, such as walking and climbing activities. Given that preliminary results were in favour of significant alterations in monitored pathognomonic symptoms, we found that it could be reasonable to verify the possible higher or lower presence of dopaminergic neuronal clusters in both the anterior and posterior parts of the *Drosophila* brain. Finally, we concluded that, at least with regard to specific lysate treatments, there was a direct relationship between the amelioration of some motor performances and the increased number of dopaminergic neurons in selective areas of *LRRK2* mutant brains.

## 2. Materials and Methods

Adult wild-types (WTs, Canton-S) and *LRRK*^WD40^ mutants (*LRRK*^ex1^ #34750, simply termed *LRRK* from Bloomington Stock Center) *Drosophila melanogaster* (*D. melanogaster*) males were used. Soon after emergence from pupae, WT and *LRRK* mutant males were separated from females. WT and mutant flies were reared on a standard cornmeal–yeast–agar medium in controlled environmental conditions (24–25 °C, 60% relative humidity, light/dark = 12/12 h). Flies ranging 10–15 days in age were tested in accordance with previous experiments [34]. In addition, groups of mutant and WT flies were reared on a standard medium supplemented with DLE from both female and male patients at two different concentrations (0.01% and 0.1%). All experiments were performed using adult flies aged 8–9 days (group I) and 15–16 days (group II), for a total of 20–40 subjects.

### 2.1. Motor Activity

The effects of human male and female DLE were assayed at 7 and 14 days of treatment at the above age steps in male *LRRK* and WTs. One day before ending the treatment, all flies from each experimental group, i.e., untreated WTs and *LRRK* and DLE-treated WTs and *LRRK*, were individually transferred to a vertically-positioned plastic tube (length 6.5 cm, diameter 0.4 cm) plugged at the bottom and with the opposite side filled with the standard cornmeal–yeast–agar medium, with or without DLE. Twenty-four hours of sleep and motor activity were recorded using the *Drosophila* Activity Monitor System (DAMS; Trikinetics, Waltham, MA, USA), and activity was determined as each fly moved back and forth in the tube and interrupted the infrared light beam that bisected each tube. Therefore, every time the fly crossed the tube was counted as movement, measured over a 1 min period. DAMS monitors were housed inside environmental chambers where temperature and humidity were kept constant.

### 2.2. Climbing Assay

The climbing assay (negative geotaxis assay) was used to assess locomotor ability [34]. Climbing data were obtained from different age groups: (I) 7–8 days old and (II) 14–15 days old, of WTs and *LRRK D. melanogaster* in basal and treated conditions. Cohorts of 30 flies from each experimental group were subjected to the assay. Flies were placed individually in a vertically positioned plastic tube (length 10 cm, diameter 1.5 cm) with the bottom plugged. Climbing time (expressed in seconds) was recorded upon crossing a line drawn 6 cm from the bottom. The number of insects that climbed to or above this line within 10 s was recorded and expressed as a percentage of total flies.

### 2.3. DLE Preparation

TF was prepared by means of buffy coats of healthy blood donors obtained from the Immunohematology Unit of Brotzu Hospital (Cagliari, Italy) upon permission of its ethics committee (registry no. 6397/2015). After several cycles of centrifugations and washes, the leukocyte pellet was resuspended in NaCl (0.9%) and immersed in nitrogen liquid. Samples were subjected to repeated cycles of freezing and thawing in order to favour the complete cellular lysis, including the plasma membranes, thereby making it possible to collect the whole extracted fraction. This content was transferred into 15 mL dialysis tubes (Slide-A-Lyzer MINI dialysis devise, 10K MW; Thermo Scientific, Bedford, MA, US) according to the manufacturer’s procedure for the recovery of proteins and macromolecules above 10 kDa and left overnight in constant agitation at room temperature. Finally, DLE obtained from both male and female patients were stored at −70 °C until experimental use.

### 2.4. Immunohistochemistry

Free-floating fluorescent immunostaining for tyrosine hydroxylase (TH) was performed on whole dissected adult brains. *LRRK2* and WT Canton *Drosophila* were anesthetised on ice before brains were rapidly dissected and fixed with paraformaldehyde (4%) in phosphate-buffered saline (PBS). After fixation and a rinse in PBS, brains were incubated with the TH primary antibody (1:100, AB152, Millipore) and normal donkey serum (10%) in PBS with Tween-20 (PBST, 0.3%) at 4 °C for 72 h. After rinsing, the brains were incubated with a donkey anti-rabbit Alexa Fluor 594 secondary antibody (1:200, Jackson ImmunoResearch) and normal donkey serum (10%) in PBST at 4 °C for 72 h. Afterwards, the brains were mounted on glass slides and coverslipped with Vectashield antifading medium. Fluorescent images were captured by a fluorescence spinning-disk confocal microscope (Crisel Instruments, Roma, Italy). Each sample was scanned through Z-stacks (63× objective). The number of TH-positive neurons of different clusters in each hemisphere of each stack was counted manually with the use of NIH ImageJ software (version 1.51i, NIH, Bethesda, MD, USA), and 10–20 hemispheres were analysed for each group of flies.

### 2.5. Statistics

All significant differences were evaluated with a two-way ANOVA and/or one-way ANOVA, followed by a post-hoc analysis when appropriate (Tukey’s test).

## 3. Results

### 3.1. Motor Activity

**Figure 1:**
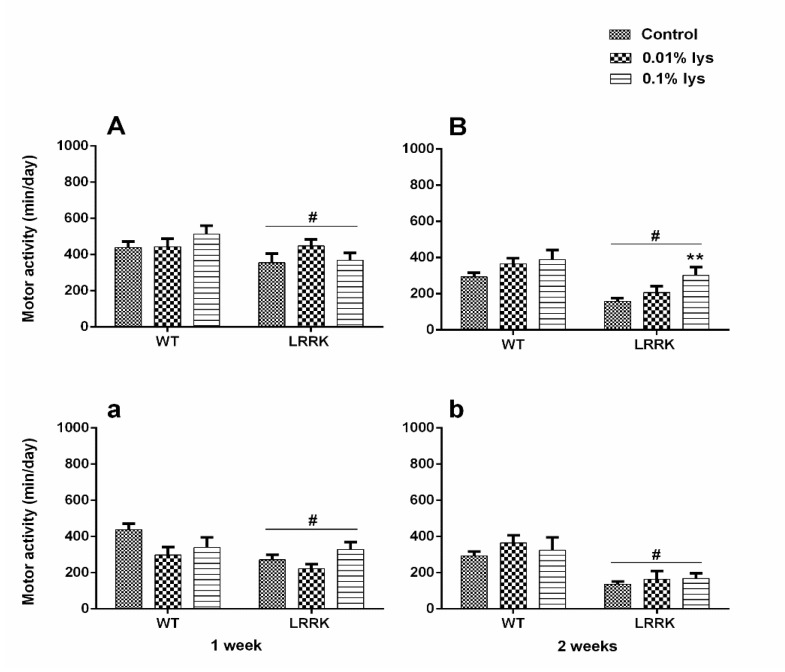
Effects of male dialyzable leukocyte extracts (mDLE) and female dialyzable leukocyte extracts (fDLE) treatments on motor activity of wild-type (WT) and *LRRK* mutant flies. Total motor activity (24 h) of WT and *LRRK* mutant flies treated with mDLE (**A**,**B**) or fDLE (**a**,**b**) in their diet at 7 days and 14 days is shown. Bars represent mean ± standard error of mean (SEM) of 20–40 fly groups in triplicate. # = *p* < 0.01 *LRRK* strain versus WT group and ** = *p* < 0.001 *LRRK* C versus *LRRK* fDLE (0.1%).

**Figure 2:**
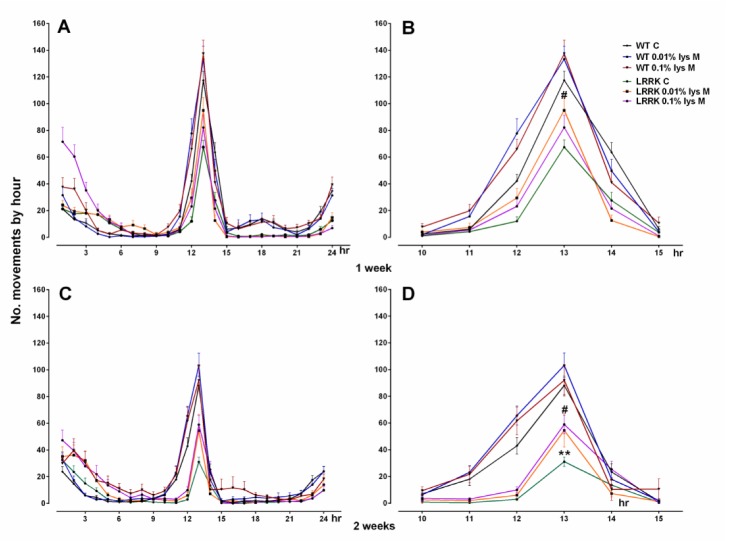
This illustrates the 5 hr and 24 hr time–course in WT and *LRRK* mutant flies treated with mDLE. # = *p* < 0.001 *LRRK* strain versus WTs and ** = *p* < 0.001 *LRKK* C versus LRRK fDLE-treated (0.01%, Tukey’s test).

As expected, a significant difference in locomotor activities between WT and *LRRK* strains was found without any variations after male DLE (mDLE) [F_(1,248)_ = 4.311, *p* = 0.0389] and female DLE (fDLE) [F_(__1,175__)_ = 6.476, *p* = 0.0118], after seven days of treatment with both doses. Neither treatment doses nor their interactions with the strains were significant (*p* > 0.05). However, data suggest a mild improvement in mutants at the highest concentration of fDLE (0.1%) and, surprisingly, at the lowest concentration (0.01%) of mDLE (Figure 1A and Figure 2, respectively).

**Figure 3:**
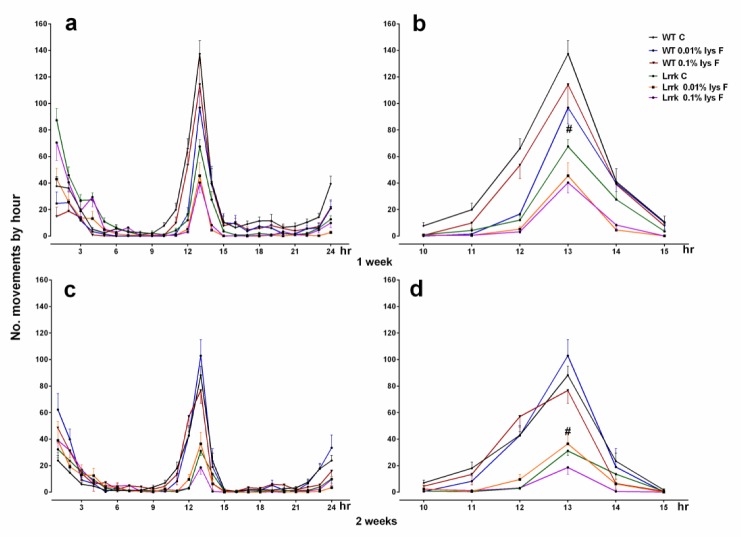
This illustrates the 5 hr and 24 hr time–course in WT and *LRRK* mutant flies treated with fDLE after 7 and 14 days of treatment. # = *p* < 0.001 *LRRK* strain versus WTs and ** = *p* < 0.001 *LRKK* C versus *LRRK* fDLE-treated (0.01%, Tukey’s test).

On the contrary, the analysis of motor activity showed a more significant difference with mDLE upon 14 days of treatment, in terms of activity performance amelioration, compared to the fDLE-treated group. Motor activity was enhanced in the flies *LRRK* group with 0.1% mDLE (*p* < 0.01). The analyses of treated and control fly groups revealed that only the most concentrated dose of mDLE was able to trigger improvements in motor activity. A two-way ANOVA found a statistically significant strain [F_(1,238)_ = 19.42, *p* < 0.0001] and treatment [F_(2,38)_ = 5.619, *p* = 0.0041] effect but not a strain × treatment interaction [F_(2,238)_ = 0.5201, *p* = 0.5952] effect indicative of overall activity (Figure 1b). A lower-order one-way ANOVA showed a significant effect of 0.1% mDLE in the *LRRK* mutants (*p* < 0.01), compared to *LRRK* control flies. The most apparent difference was detected in the *LRRK* group at two weeks with both doses [F_(1,143)_ = 35.63, *p* < 0.0001] (Figure 1b). Conversely, the WT flies treated with both mDLE and fDLE (0.01% and 0.1%) did not show any significant drug effects on motor activity compared to untreated controls (Figure 1A,B, Figure 2 and Figure 3, respectively). For the group treated with fDLE, the two-way ANOVA found a statistically significant effect only for the strain [F_(1,143)_ = 35.63, *p* < 0.0001) but not for treatment [F_(2,143)_ = 0.9761, *p* = 0.3793] or treatment × strain [F_(2,143)_ = 0.2869, *p* = 0.7510]. The time–course activity measured for 24 h at 7 and 14 days is illustrated in Figure 2A,C for mDLE and Figure 3a,c for fDLE at 7 days, respectively. Interestingly, there was a common feature during the whole observed period represented by a peak in motor activity from the 10 to 15-hours interval in both strains, with specific significant values always expressed at 13 hr (Figure 1B,b and Figure 3b,d, respectively at 14 days). In the Supplementary Material, Data in Figure S1 are represented as box whisker graphs for more transparency in showing their variability.

### 3.2. Climbing Activity

**Figure 4:**
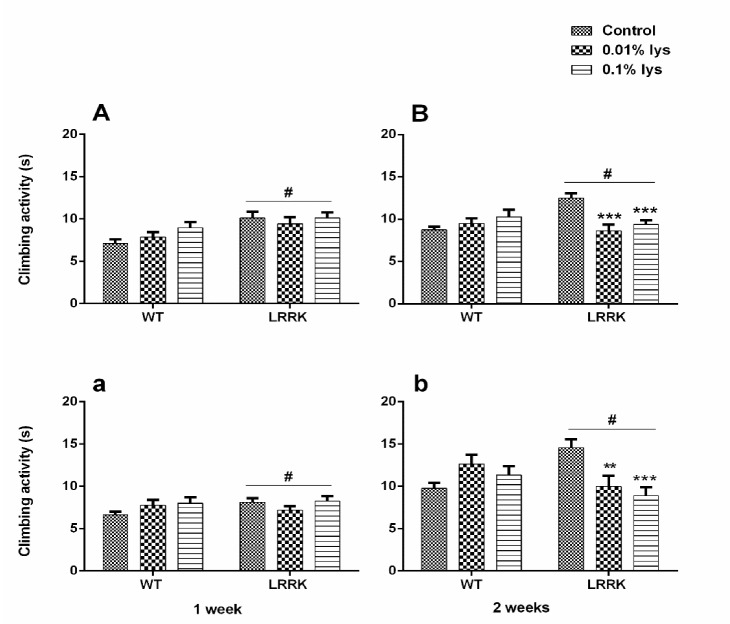
Effects of mDLE and fDLE treatments on climbing activity of mutant WT and *LRRK* flies, with mDLE (**A**,**B**) and fDLE (**a**,**b**) in their diet at 7 (**A**,**a**) and 14 (**B**,**b**) days of treatment, respectively. Bars represent mean ± SEM of 20–40 fly groups in triplicate. # = *p* < 0.05 WTs versus *LRRK* in (**A**), (**a**,**b**) groups; ** = *p* < 0.01 *LRRK* versus WTs control and WT mDLE (0.01%) and *** = *p* < 0.0001 *LRRK* versus *LRRK* (0.01%) and mDLE (0.1%), respectively. ** = *p* < 0.01 *LRRK* versus *LRRK* (0.01%) and *** = *p* < 0.0001 *LRRK* versus *LRRK* fDLE (0.1%, Tukey’s test).

At only seven days, a statistically significant strain difference in climbing was observed independently in mDLE [F_(1,479)_ = 13.05, *p* = 0.0003] and fDLE [F_(1,476)_ = 7.178, *p* = 0.0076] treatments (Figure 4A,a). Moreover, after 14 days, a significant increase in restored climbing performance was detected in control (nontreated) mutant *LRRK* group, suggesting a motor disability recovery (Figure 4B,b). In particular, at two weeks, a statistically significant strain × treatment interaction [F_(2,476)_ = 1.944, *p* = 0.1443] and treatment-dependent effect [F_(2,794)_ = 3.354, *p* = 0.0354] (two-way ANOVA) was observed without any significant strain [F_(1,794)_ = 1.815, *p* = 0.1782] effect. However, a lower-order one-way ANOVA showed a significant effect of mDLE at both 0.01% and 0.1% concentrations for the *LRRK* mutants (*p* < 0.05), compared to WT control flies, where there was no treatment effect (Figure 4). Similarly, fDLE induced better performances when administered at 14 days, with a significant strain × treatment interaction [F_(2,449)_ = 9.096, *p* = 0.0001] but no strain [F_(1,449)_ = 0.01557, *p* = 0.9008] or treatment effect [F_(2,449)_ = 2.141, *p* = 0.1187] due to the significant differences in variance within the groups (Bartlett’s test, *p* = 0.0001; Figure 4b). Hence, after a lower-order one-way ANOVA, the differences between strain and treatment were evident and significant [F_(4,601)_ = 8.469, *p* < 0.0001] (Figure 4b). In the Supplementary Material, Figure S2 illustrates the same data as whiskers graphs for more transparency in showing their variability.

### 3.3. Immunohistochemistry

**Figure 5:**
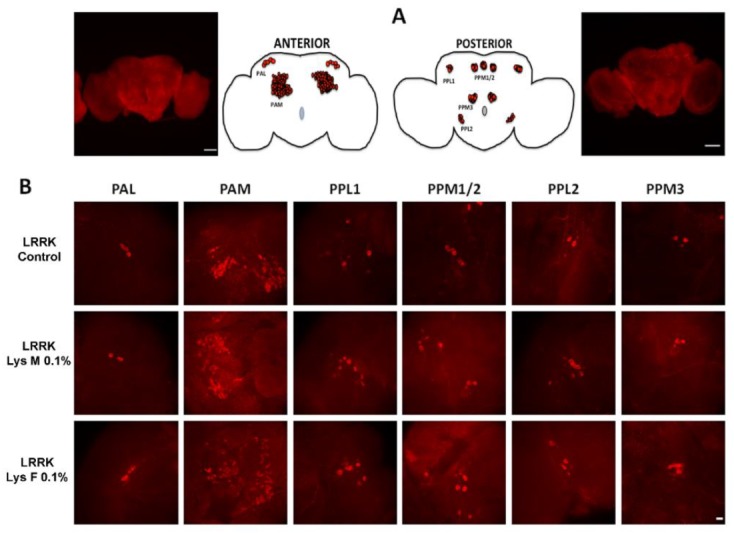
Anterior and posterior dopaminergic (DA) clusters in *LRRK* flies. A = micrograph (10×) and schematic representation of brain anterior and posterior faces where the DA clusters reside. B = representative image stacks (63×) showing the distribution of dopaminergic neurons in the different clusters in *LRRK* control and mDLE (LysM) and fDLE (LysF)-treated groups. The bars represent 100 (Panel A) and 10 µm (Panel B).

**Figure 6:**
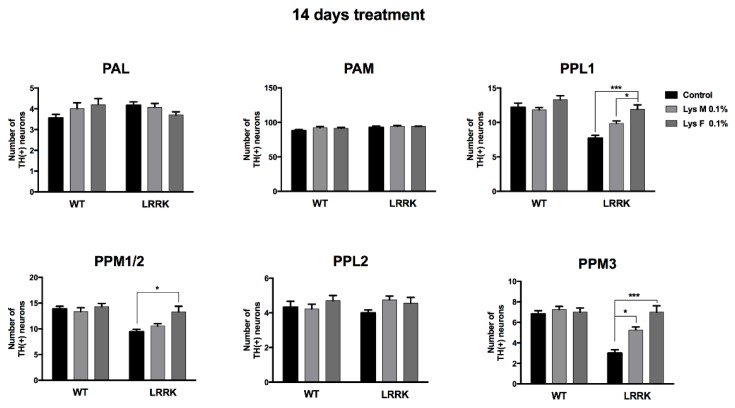
Effects of mDLE and fDLE treatments on neuron numbers in DA clusters of WT and *LRRK* flies. Graphs show no differences in the number of DA neurons in protocerebral anterior medial (PAM) and protocerebral anterior lateral (PAL) of the different groups. The treatments with mDLE and fDLE at 14 days significantly prevent the loss of DA neurons in the posterior clusters (protocerebral posterior lateral (PPL)1, PPL2, and protocerebral posterior medial (PPM)1/2 and PPM3) of *LRRK2*^WD40^. * = *p* < 0.05; *** = *p* < 0.001 in control groups (10 hemispheres for each group, Tukey’s test).

As shown in Figure 5, we focused on the anterior dopaminergic (DA) clusters, protocerebral anterior medial (PAM) and protocerebral anterior lateral (PAL), on the posterior clusters, protocerebral posterior laterals (PPL1 and PPL2) and protocerebral posterior medial: superior-medial/inferior-medial (PPM1/2 and PPM3) in order to ascertain any possible number variations triggered by mDLE and fDLE after 14 days, since the physiological parameters described above were significantly recovered by this timepoint. The *LRRK* flies showed a reduction of DA neurons with respect to WT groups in all posterior clusters. In particular, for the PPL1, there was a significant strain [F_(2,64)_ = 15.83, *p* < 0.0001], treatment [F_(1,64)_ = 45.03, *p* < 0.0001] effect and strain × treatment interaction [F_(2,64)_ = 5.893, *p* = 0.0045], while in the PPM1/2, only significant strain [F_(1,58)_ = 24.91, *p* < 0.0001] and treatment [F_(2,58)_ = 5.942, *p* = 0.0045] effects were found but not a strain × treatment interaction [F_(2,58)_ = 31.40, *p* = 0.0507]. The treatment with mDLE and fDLE after 14 days significantly prevented the loss of DA neurons in the posterior clusters PPL1, PPM3 and PPM1/2 of *LRRK*. The only cluster not responsive to the DLE effects was the PPL2 (strain: [F_(1,49)_ = 0.005574, *p* = 0.9408]; treatment: [F_(2,49)_ = 1.437, *p* = 0.2475] and strain × treatment: [F_(2,49)_ = 1.316, *p* = 0.2774]). In particular, fDLE seemed to always be more successful for neuronal rescue compared to the mDLE, and in the case of the PPM1/2 cluster, only fDLE displayed an upregulation of DA neurons. Moreover, the PPL1 showed significant strain [F_(2,64)_ = 15.83, *p* < 0.0001]; treatment [F_(1,64)_ = 45.03, *p* < 0.0001] effect and strain × treatment interaction [F_(2,64)_ = 5.893, *p* = 0.0045], while in the PPM1/2, only significant strain [F_(1,58)_ = 24.91, *p* < 0.0001] and treatment [F_(2,58)_ = 5.942, *p* = 0.0045] effect but not strain × treatment interaction [F_(2,58)_ = 3140, *p* = 0.0507]. PPM3 showed strain [F_(1,47)_ = 32.92, *p* < 0.0001], treatment [F_(2,47)_ = 12.93, *p* < 0.0001] effect and strain × treatment interaction [F_(2,47)_ = 10.67, *p* = 0.0002] (Figure 6). No differences were found in the anterior clusters PAM and PAL. In the Supplementary Material, Figure S3 represents the anterior and posterior DA clusters in WT flies (A). Representative image stacks (63×) show the distribution of dopaminergic neurons in the different clusters in WT control flies. The bars represent 100 (Panel A) and 10 µm (Panel B).

## 4. Discussion

It is well-established that mutations in the *LRRK2* gene are the most frequent cause of familial PD [41,42]. Clinically, mutant *LRRK2*-PD patients are often considered indistinguishable from sporadic patients. Likewise, *LRRK2* is also highly recruited in immune cells and its variability is under the control of immune stimulation, providing compelling evidence that both systemic and central nervous system (CNS) inflammations interact in PD pathophysiology [43]. As a matter of fact, the human DLE used in this study as a potential “transfer factor” of cellular-mediated immunity opens up intriguing scenarios for controlling and interpreting the progression of PD. Moreover, despite unknown molecular mechanisms, specific signalling pathways for cellular survival and activation involve the *D. melanogaster* Toll protein, which is homologous to the receptors for human IL-1 (proinflammatory cytokine) [44]. Hence, it is feasible that selected and specific DLE molecules that are prone to binding Toll-like receptors have recently been regarded as alternative therapy in PD [45]. In principle, the overall analyses of activities have shown that mutant *LRRK* flies require a longer period (two weeks) of DLE treatment in order to trigger motor and climbing performances similar to WT flies. This result was observed in climbing activity irrespective of the DLE origin (male or female). Only for motor activity was the mDLE more effective at the highest dose, compared to the fDLE. Hence, in our search for anatomical changes indicative of motor function improvements, we measured with immunohistochemistry the number of TH-positive neurons in the anterior and posterior clusters highly related to motor ability. In particular, the dopaminergic PPM3 cluster projects its fibres into the central complex, which is involved in controlling locomotion and arousal [46]. The immunohistochemical results were in agreement with the above hypothesis, but only posterior clusters, including PPL1 and PPM1/2, were involved with the upregulation of DA neurons. Most notably, the fDLE (0.1%) were always more effective in promoting the increase of DA neurons, compared to the mDLE. In conclusion, this is the first paper that has investigated the positive effects of DLE in sustaining motor parameters severely affected in mutant *LRKK* by rescuing those DA clusters that are highly related to motor ability. Finally, we can also speculate whether more clear-cut results could be generated in a different dose-dependent experimental setting as a direct reflection of inflammatory imbalances in pro- and antiinflammatory molecules [47]. For that purpose, experiments are in progress to analyse the biochemical composition of DLE from male and female subjects in order to ascertain possible molecular alternatives to be recruited, not only for immunomodulation but also for neuroprotective interventions.

## Supplementary Materials

The following are available online at https://www.mdpi.com/2076-3425/10/1/45/s1: Figure S1, Motor activity of WT and LRRK mutant flies treated with mDLE (A-B) and fDLE (a-b) in their diet at 7 days (A-a) and 14 days (B-b) during 24-h daytime. The top and bottom of the box and whisker plots show the upper and lower quartiles, respectively. The horizontal line in the middle indicates the median of the corresponding distribution, while the minimum and maximum observed values are indicated by the bars connected to the box. #, P < 0.001 versus the WT strain; Figure S2, Climbing activity of WT and LRRK mutant flies treated with mDLE (A-B) and fDLE (a-b) in their diet at 7 days (A-a) and 14 days (B-b) during 24-h daytime. The top and bottom of the box and whisker plots show the upper and lower quartiles, respectively. The horizontal line in the middle indicates the median of the corresponding distribution, while the minimum and maximum observed values are indicated by the bars connected to the box. #, *P* < 0.001 versus the WT strain; ***, *P* < 0.0001 LRRKC vs LRRK 0.01% and 0,1% mDLE, and 0.1% fDLE, respectively. **, *P* < 0.001 LRRKC vs LRRK 0.01% fDLE; Figure S3, Anterior and posterior DA clusters in WT flies. Distribution of Anterior and posterior DA clusters (A); Representative image stacks (63X) showing the distribution of dopaminergic neurons in the different clusters in WT control flies. The bars represent 100 (Panel A) and 10 µm (Panel B).
