# Supplementary material for: Improvements of Motor Performances in the Drosophila LRRK2 Loss-of-Function Model of Parkinson’s Disease: Effects of Dialyzed Leucocyte Extracts from Human Serum
Source: Brain Sci. 2020 Jan 14;10(1):45. doi: 10.3390/brainsci10010045 (PMC7017078; doi:10.3390/brainsci10010045)
Supplement: Supplementary file 1 [file brainsci-10-00045-s001.pdf]

Supplementary Material: Improvements of Motor Performances in the *Drosophila LRRK2* Loss-of-Function Model of Parkinson's Disease: Effects of Dialyzed Leucocyte Extracts from Human Serum

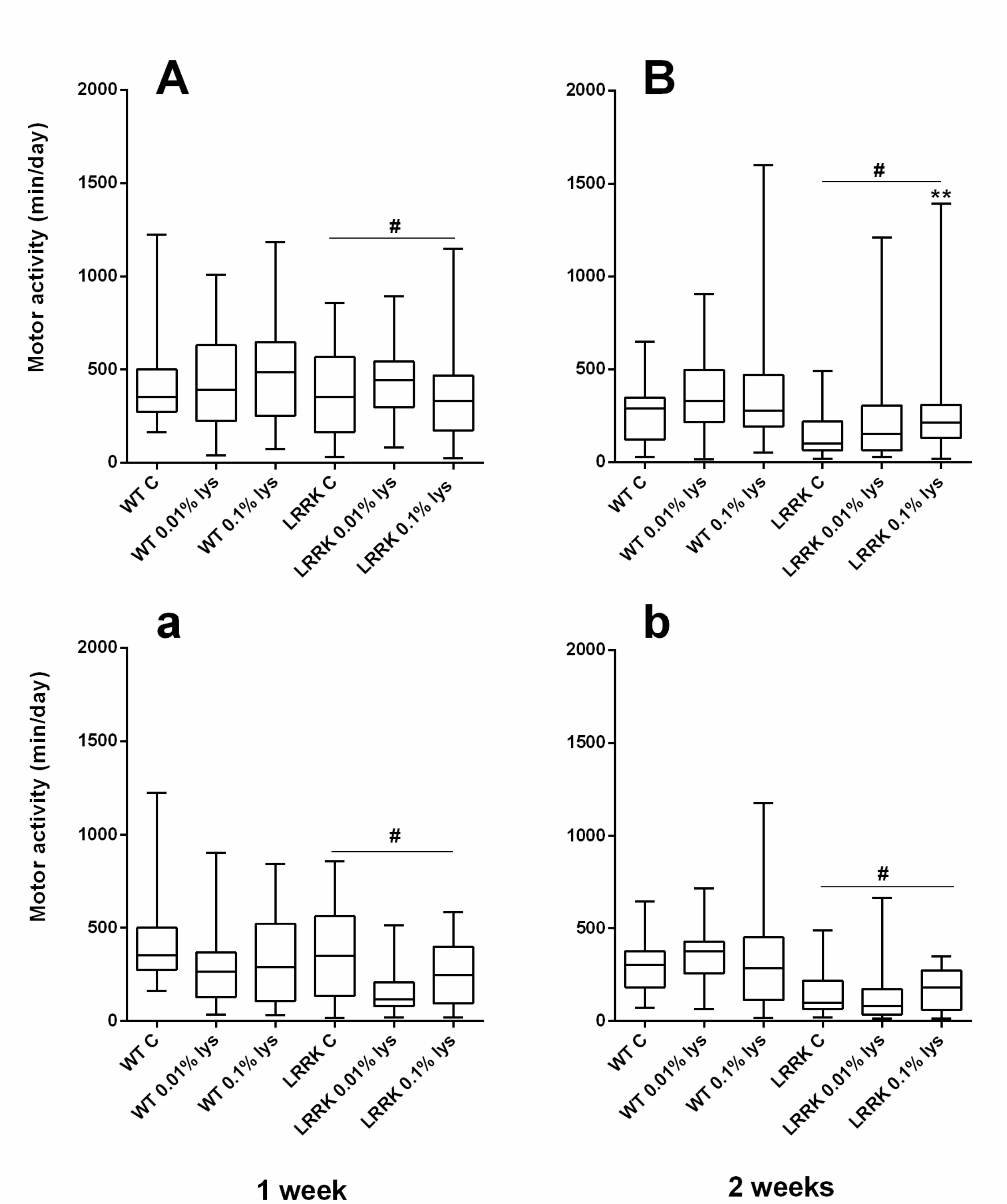

Figure S1. Motor activity of WT and LRRK mutant flies treated with mDLE (A-B) and fDLE (a-b) in their diet at 7 days (A-a) and 14 days (B-b) during 24-h daytime. The top and bottom of the box and whisker plots show the upper and lower quartiles, respectively. The horizontal line in the middle indicates the median of the corresponding distribution, while the minimum and maximum observed values are indicated by the bars connected to the box. #, $P < 0.001$ versus the WT strain.

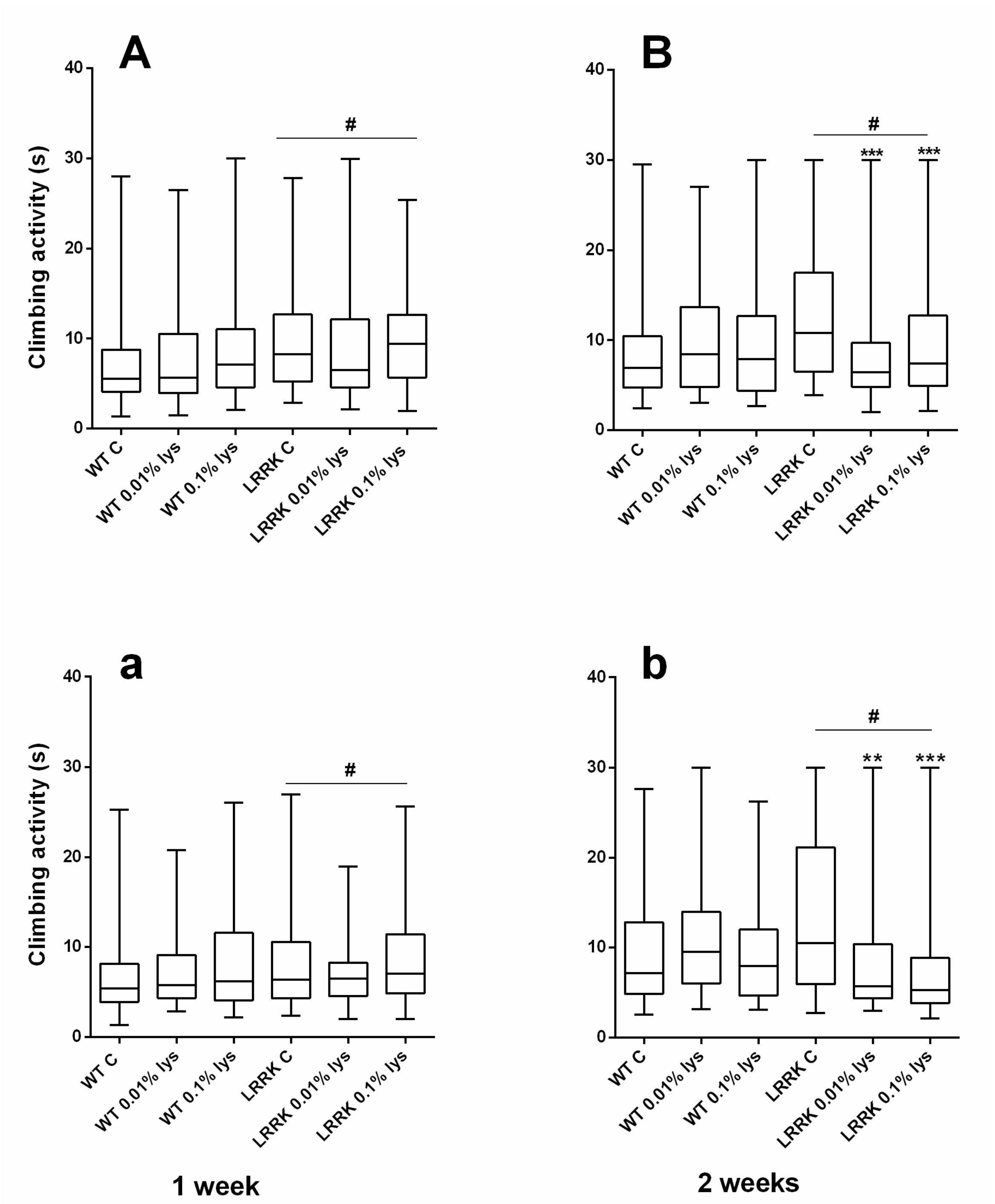

Figure S2. Climbing activity of WT and LRRK mutant flies treated with mDLE (A-B) and fDLE (a-b) in their diet at 7 days (A-a) and 14 days (B-b) during 24-h daytime. The top and bottom of the box and whisker plots show the upper and lower quartiles, respectively. The horizontal line in the middle indicates the median of the corresponding distribution, while the minimum and maximum observed values are indicated by the bars connected to the box. #, $P < 0.001$ versus the WT strain; ***, $P < 0.0001$ LRRKC vs LRRK 0.01% and 0,1% mDLE, and 0.1% fDLE, respectively. **, $P < 0.001$ LRRKC vs LRRK 0.01% fDLE.

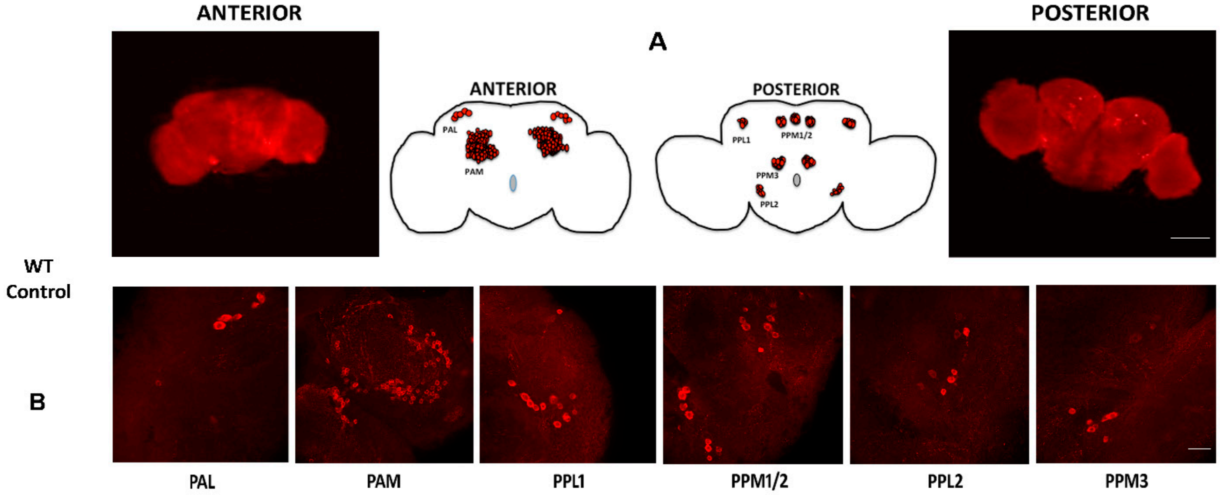

Figure S3. Anterior and posterior DA clusters in WT flies. Distribution of Anterior and posterior DA clusters (A); Representative image stacks (63X) showing the distribution of dopaminergic neurons in the different clusters in WT control flies. The bars represent 100 (Panel A) and 10 μm (Panel B).
